# Editorial: Emerging and old viral diseases: Antiviral drug discovery from medicinal plants

**DOI:** 10.3389/fphar.2022.976592

**Published:** 2022-08-19

**Authors:** Mohammed Rahmatullah, Rownak Jahan, Veeranoot Nissapatorn, Maria De Lourdes Pereira, Christophe Wiart

**Affiliations:** ^1^ Department of Biotechnology and Genetic Engineering, Faculty of Life Sciences, University of Development Alternative, Dhaka, Bangladesh; ^2^ School of Allied Health Sciences and World Union for Herbal Drug Discovery (WUHeDD), Walailak University, Nakhon Si Thammarat, Thailand; ^3^ CICECO-Aveiro Institute of Materials and Department of Medical Sciences, University of Aveiro, Aveiro, Portugal; ^4^ School of Pharmacy, University of Nottingham Malaysia Campus, Selangor, Malaysia

**Keywords:** viruses, molecular docking, coronavirus, Bromelain, traditional methods

It has been mentioned in a number of studies that viruses and viral diseases may have had a profound effect on human evolution, culture, and civilization. The type of viruses affecting human beings have changed with time, depending on human density and climatic conditions (de S Leal and Zanotto, 2000; Sharp and Simmond, 2011). The human genome is considered by scientists to be not only a hereditary blue-print, but also as a niche colonized by numbers of endogenous retrovirus-derived retroposons and other mobile genetic elements (de Koning et al., 2011). When and how this started remains an unanswered question, as to other questions like whether such viral interference of human genomes or viral diseases affecting humans came in distinct waves or were simply a part of gradual infiltration amidst continual interactions between viruses and humans.

Irrespective of the past, recent decades have witnessed emergence of dozens of new viruses and re-emergence of old viruses, a number of them being zoonotic. Zoonotic viral diseases are transmitted from a host animal harboring the virus to humans, occasionally through a secondary host. The rise in incidences of new zoonotic viral diseases can be attributed to a number of factors including live animal markets, wildlife hunting and consumption, intensive farming of domestic animals and wildlife, and last but not the least—increases in human habitat and agricultural land, leading to massive deforestation of forests with concomitant increases in human-wildlife contacts (Wolfe et al., 2005; Magouras et al., 2020). It has also been suggested that global climate changes (due to human-caused exorbitant atmospheric increases in carbon dioxide and greenhouse gases like methane) are also causative factors behind the incidences of emerging infectious diseases (Patz et al., 1996; Vora, 2008). Carlson et al. (2022) pointed out that currently at least 10,000 virus species possess the capacity to infect humans, but the vast majority are circulating among wild animals without affecting humans; the authors conclude that climate change will increase cross-species transmission of virus by about 4,000 times. Although all viruses may not spread to humans, the possibility of near-pandemics and an actual pandemic caused by the three coronaviruses, Severe Acute Respiratory Syndrome Coronavirus (SARS-CoV), Middle East Respiratory Syndrome Coronavirus (MERS-CoV), and Severe Acute Respiratory Syndrome Coronavirus 2 (SARS-CoV-2) are possible harbingers of things to come.

Changing pattern of arthropod distribution due to climate change was possibly the reason for introduction of the bluetongue virus (serotype 8) in northwestern Europe in 2005/2006. Arenaviruses, bunyaviruses, hantaviruses and noroviruses have also shown emergence evidences in the recent past, and climate change may have been a major factor behind this first reported emergence (Gould, 2009). Dislocation of people because of food shortages and overcrowding in places have been mentioned as the causative factor behind the increase and spread in arboviruses like dengue, yellow fever, and West Nile virus (Schvoerer et al., 2008). It is expected that because of climate changes, the distribution of disease-causing vectors will change, leading to an increase in old and new bacterial, viral, and parasitic diseases (El-Sayed and Kamel, 2020). The world has gotten globalized with an immense increase in travel of people and goods; this factor is the single most conducive factor behind the rapid spread of diseases in a very short time, the most recent example being the spread of monkey pox. Since May 13 till 2 June 2022, the number of confirmed cases reported to the World Health Organization (WHO) reached 780, and the disease has spread to 27 member States across four WHO regions that are not endemic for monkeypox virus (WHO, 2022a).

As will be discussed with relevance to the papers published in this special issue, the destruction of tropical rain forests and so for that matter other types of forests and plant species are exacerbating the global climate change and forming a vicious cycle. Plants store carbon dioxide, and deforestation leads to release of the stored carbon dioxide back to the atmosphere. This causes an increase in temperature and variations in rainfall (even desertification), which can become a cause for greater loss of plant species (Tariq, 2015). Cattle ranchers and rainforest clearing, for instance, goes hand in hand in the Amazons (Skidmore et al., 2021); conversion of previous rainforest and other areas to cattle farming is one of the largest anthropogenic causes behind the increase of the greenhouse gas methane in the atmosphere for cattle produce prodigious amounts of methane (Moumen et al., 2016). But what is of more relevance for the present issue is the loss of medicinal plant species, which is also seeing an increasing loss in diversity because of unfavorable weather conditions due to global warming and the clearance of forest areas and fallow lands for agricultural activities to feed the burgeoning population of the planet and the adoption of more meat in dietary food habits. All plants produce secondary metabolites, which phytochemicals have diverse pharmacological activities. The pharmacological activities may range from beneficial to toxic, or beneficial at low doses and turning toxic at high doses. Plants have been used for medicinal purposes since antiquity and possibly from the advent of human beings (Conceição et al., 2020). Even as of now, a number of conventional drugs (morphine, artemisinin, paclitaxel to name only three) have been discovered on the basis of medicinal plants studies and/or close observations of traditional medicinal practices (Najmi et al., 2022).

So what is the scenario we are looking at? To take the example of SARS-CoV-2-caused COVID-19 pandemic, as of now, we still do not have an approved conventional anti-viral drug for this disease, which can work with all COVID-19 patients at all stages of the disease and is basically free of adverse effects. On 22 April 2022, WHO strongly recommended nirmatrelvir and ritonavir, but only for mild and moderate COVID-19 patients (WHO, 2022b). It is also not certain whether low to moderate-income group patients will be able to afford this anti-viral combination drug. Also to be noted is that COVID-19 emerged in December 2019, and as of 12 June 2022 the pandemic has been the cause of 540,312,821 infections and 6,331,211 deaths worldwide (https://www.worldometers.info/coronavirus/). A number of vaccines against COVID-19 have emerged. Although a lot of ingenuity and hard work has gone behind the development of these vaccines, their efficacies have already been compromised with the emergence of newer “variants” of SARS-CoV-2, like the Omicron variant against which vaccine efficacy waned even after two doses followed by a booster dose (Andrews et al., 2022). And this is only one viral disease. Even for coronaviruses, one does not know whether SARS and MERS, which disappeared as suddenly as they came, might as suddenly re-emerge in a new variant form, which will be more transmissible with an even higher mortality rate (MERS had a 34% mortality rate).

The absence of effective vaccines against COVID-19 (of the small pox or polio-type) and lack of conventional drugs is compelling researchers, academicians, scientists, and other stakeholders (like WHO) to give traditional formulations (mostly plant-based) and phytochemicals a closer look to find effective remedies against COVID-19, as well as other viral diseases. Interestingly, the seven articles, which made up the first volume of the series “**Emerging viral diseases: Antiviral drug discovery from medicinal plants**” all dealt with medicinal plants or plant products with the primary emphasis on SARS-CoV-2. This is a strong point of the issue; since the articles mainly converged on SARS-CoV-2 therapeutics, there is a hope that something positive in the nature of therapeutics against SARS-CoV-2 might come out from the literature. The main drawback of the articles is that though some new viruses like Ebola were mentioned, there was a lack of elaborate discussion on possible prophylactic/therapeutic on other emerging viruses like Hendra, Nypah, Hanta, Junin, or West Nile, to name only a few.

Africa is a continent with 3,000 tribes. The enormous variety of traditional/tribal medicines in Africa allow one to think that a number of positive outcomes can come out from a systematic analyses of Traditional African Medicine (TAM), which at the moment is more noted in people’s minds for its animistic beliefs, divinations and other exotic practices (voodoo being a prime example), and administration of plant and animal parts for treatment. Recognizing the richness of TAM but the scarcity of available literature on TAM systems, WHO is also encouraging development of TAM and using TAM as a substitute for conventional medicines (Rasamiravaka et al., 2015). The continent has over 45,000 plant species, which makes it richer in the number of plants than other countries with more recognized Traditional Medicinal Systems (TMS) like China and India.

In their article, Beressa et al. made a comprehensive study of African antiviral medicinal plants and makes a strong point for their potential values of therapeutic uses and discovery of antiviral components. The authors examined 36 studies and screened a total of 328 plants of which 127 plants were deemed to be active against 25 viral species. A number of viruses were included in their review including old viruses like the polio virus, and emerging viruses like SARS-CoV-2, Zika and Ebola viruses. Several phytochemicals were reported to be active in molecular docking studies with the main protease, Mpro/3CLpro of SARS-CoV-2. Some of these reported compounds were 6-oxoisoiguesterin, crocin, digitoxigenin, and β-eudesmol (the latter three in Moroccan medicinal plants). Another *in silico* study aimed to identify SARS-CoV-2 inhibitors from South African medicinal plants. Arabic acid and L-canavanine were found to be potential inhibitors of SARS-CoV-2 Mpro (Dwarka et al., 2020). Although thus far, none of the compounds have gone through clinical trials and obtained approval for therapeutic uses against COVID-19, the differences in the anti-SARS-CoV-2 compounds from northern African (Morocco) versus southern African (South Africa) plants is an indicator of the richness of the plant kingdom in yielding potential antiviral compounds only if proper searches are carried out. The same richness is also visible in a report from West Africa, where citrus fruits from West Africa containing quercetin, hesperitin, naringenin, nobiletin and tangeretin were found *in silico* studies to be potential inhibitors of Mpro of SARS-CoV. The sulfoxide, alliin, a natural component of fresh garlic was found to be a potential inhibitor of SARS-CoV-2 Mpro (Popoola et al., 2022).

Bachar et al. published their article on antiviral plants of Bangladesh and provided insights into their bioactive metabolites on SARS-CoV-2, human immunodeficiency virus (HIV), and hepatitis B virus (HBV). Their review covers 46 antiviral medicinal plants and 79 bioactive metabolites with antiviral properties. The authors pointed out that among the metabolites, “hesperidin, apigenin, luteolin, seselin, 6-gingerol, humulene epoxide, quercetin, kaempferol, curcumin, and epigallocatechin-3-gallate (EGCG) have been reported to inhibit multiple molecular targets of SARS-CoV-2 viral replication in a number of *in silico* investigations”. Although the SARS-CoV-2 investigations involved only computational assays, *in vitro* and *in vivo* assays along with *in silico* studies demonstrated the anti-HIV activities of EGCG, anolignan-A, and B, ajoene, curcumin, and oleanolic acid. Anti-HBV activities were exhibited by piperine, ursolic acid, oleanolic acid, (+)-cycloolivil-4′-*O*-β-dglucopyranoside, quercetin, EGCG, kaempferol, aloin, apigenin, rosmarinic acid, andrographolide, and hesperidin. Interestingly, flavonoid compounds appear to play a major role as anti-SARS-CoV-2 compounds, as seen in both West Africa as well as Bangladesh COVID-19 traditional treatments and *in silico* studies. In fact, flavonoids have been described as a complementary approach to conventional treatment of COVID-19, for *in silico* studies have shown these compounds to be potential inhibitors of Mpro of SARS-CoV, MERS-CoV, and SARS-CoV-2 (Solnier and Fladerer, 2021). However, two major questions remain about the suitability of flavonoids and the results obtained with them in even virucidal studies. For instance, one of the most reported antiviral flavonoids, quercetin, form large aggregates and so it is binding to Mpro or other proteins may reflect non-specific binding (Pohjala and Tammela, 2012). The second question concerns the bioavailability of flavonoids and a number of methods have been proposed to increase flavonoid bioavailability (Zhao et al., 2019).

However, in their manuscript, the authors Bachar et al. did an excellent job in detailing not only plants, but also plant parts and bioactive components of the plants, which have been found to give antiviral activity against different viruses. A more detailed analysis of the limitations of the compounds (like toxicity, availability, synthetic possibility) would have added more valuable information. Also, more information/discussion could have proved useful, particularly for *in silico* studies on large compounds like (+)-pinoresinol 4-*O*-(6”-*O*-vanilloyl)-β-D-glucopyranoside 6′-*O*-vanilloyltachioside 6′-Ovanilloyl-isotachioside from *Calotropis gigantea* (L.) Dryand. In Table 1 of their paper, a delineation of which compound acts against which virus would have made the otherwise excellent Table more legible to the readers. To get this information, one has to proceed onto Table 3.

Also plants like *Mangifera indica* L., and *Azadirachta indica* A. Juss. (to name only two plants) are found in a number of other countries, where among other medicinal uses, they also serve as antiviral plants (Umar et al., 2021). The buds of *M. indica* are used in Burkina Faso to control COVID-19. *Carica papaya, Azadirachta indica*, *Allium cepa*, and *Allium sativum* are common plants of Bangladesh used to treat diverse virus infections but used in Burkina Faso as treatment of COVID-19 (Babili et al., 2021). Chyawanprash and Ashokarishta are two well-known herbal formulations in the Ayurveda system of medicine in India containing *Cyperus rotundus* as one of the ingredients (Kamala et al., 2018). Chyawanprash has been used since ancient times as a health supplement and to boost immunity and longevity (Sharma et al., 2019); these properties are necessary components for particularly elderly COVID-19 patients. What comes out is that antiviral plants of Bangladesh are present and have traditional antiviral uses in other countries and other societies and more and more scientific studies are validating their traditional uses. This is a line of thought that requires further exploration, and while that is not the object of this editorial, we take this opportunity to point out that similarity in ethnomedicinal uses in different societies would strongly suggest the value of such uses in discovery of lead compounds and/or novel drugs. It is also noteworthy that little ethnomedicinal data is available from countries like Myanmar, Laos, Cambodia, or even Thailand. Comparative analyses of traditional uses of medicinal plants from these countries along with Malaysia, Indonesia and sub-Saharan African countries with traditional uses of medicinal plants in Bangladesh, India, and Pakistan has assumed greater importance in the present days of emerging pathogens.

SARS-CoV-2 is characterized by its ability to produce variants, which are more lethal or more adept at negating existing vaccine efficacies. In a small but densely populated country like Bangladesh, there is already evidences of the presence of the B.1.1.7 (Alpha) variant from the United Kingdom, the B.1.351 (Beta) variant from South Africa, the P.1 (Gamma) variant from Brazil, and the B.1.617 (Delta) variant from India (Hasan et al., 2021), and lately, the Omicron (B.1.1.529) variant (Islam, 2022). Tallei et al. has filled an important niche in showing through molecular docking and molecular dynamics studies the importance of bromelain to counteract the various variants of SARS-CoV-2.

Pineapples contain a group of digestive enzymes known as bromelains. A bromelain-derived peptide DYGAVNEVK has been shown in another study to interact with several critical receptor binding domain (RBD) residues of the SARS-CoV-2 spike glycoprotein, which is responsible for binding to its human angiotensin converting enzyme 2 (hACE2) receptor. Moreover, this interaction has been shown in computer simulation studies to occur with RBD of variants of SARS-CoV-2 (Tallei et al., 2022). A combination of bromelain and acetylcysteine (BromAc) reportedly inactivated SARS-CoV-2 in *vitro* whole virus culture of both wild-type and spike mutants (Akhter et al., 2021). Bromelain is also useful for its antimicrobial and anti-inflammatory properties (Onken et al., 2008; Ali et al., 2015).

Two papers in this special issue dealt with Traditional Chinese Medicines (TCMs) for treatment of COVID-19. As far as traditional medicines go, TCM has always held a fascination for researchers due to the vastness of its literature, number of plants used in the various formulations, and the large number of diseases treated. The nearest equivalent to TCM is Ayurveda in India. Relevant information on 14 Chinese Patent Medicines (CPMs) were collected and discussed as to their possible efficacies in treatment of COVID-19. These patented formulations, according to the authors, included “Huoxiangzhengqi capsules (pills, liquid, oral solution), Jinhuaqinggan granules, Lianhuaqingwen capsules (granules), Shufengjiedu capsules, Xiyanping injections, Xuebijing injections, Reduning injections, Tanreqing injections, Xingnaojing injections, Shenfu injections, Shengmai injections, Angongniuhuang pills, and Suhexiang pills”. The conclusion drawn from the study was that these CPMs when used with conventional medicines were effective in managing COVID-19 (Wu and Zhong).

Interestingly, in the six oral CPMs, four (Huoxiangzhengqi Capsule/Pill/Oral liquids, Jinhuaqinggan Granules, Lianhuaqinwen Capsule/Granules, and Shufengjiedu Capsule/Granules) contained *Glycyrrhiza uralensis* Fisch. ex DC among other ingredients. The plant and especially its roots/rhizomes are known to contain glycyrrhizin (Ji et al., 2016); the efficacy of glycyrrhizin (glycyrrhizic acid, a triterpene glycoside) against COVID-19 will be dealt with later in another paper published in this issue. Glycyrrhizin/glycyrrhizic acid is a common component of licorice, which is another name for *Glycyrrhiza glabra* L. (Fabaceae) and has been used in TCM and Ayurveda for thousands of years as treatment for respiratory diseases and intestinal disorders (Joshi, 2014; dos Santos Leite et al., 2022). In two oral formulations, Jinhuaqinggan Granules and Lianhuaqinwen Capsule/Granules contained *Lonicera japonica* Thunb. (Caprifoliaceae), a plant also reported by others to be beneficial for COVID-19 treatment (An et al., 2021). The parts of the plant used in the herbal formulations in Table 1 could have added extra clarity to the paper. However, the flower (flos) is usually the most used part and flowers are known to contain flavonoid compounds like quercetin, rutin, and luteolin (Li B. et al., 2020); in general flavonoid compounds, and in particular these three flavonoid compounds have shown activity against SARS-CoV-2 (Agrawal et al., 2021; Shawan et al., 2021; Solnier and Fladerer, 2021). Although this is not the proper forum for discussion of flavonoids and their anti-SARS-CoV-2 potentials, it can be said that the largest number of compounds attracting the attention of scientists against SARS-CoV, MERS-CoV, and SARS-CoV-2 belonged to the flavonoid group (Russo et al., 2020).

Suhexiang pills and Angongniuhuang pills, as pointed by the authors, contain cinnabar (HgS) and realgar (As_2_S_2_), that is, sulfides of mercury and arsenic, respectively. That leads to the question as to when and for how long these formulations can be used, or used at all. Use of heavy metals and arsenic is not unique to just these two TCM formulations or even unique to TCM. As per Chinese Pharmacopoeia, at least a third of the herbal formulations contained toxic metals in the form of cadmium, lead, arsenic, mercury, and copper (Luo et al., 2020). A study conducted in Nigeria found toxic concentrations of Fe, Ni, Cd, Cu, Pb, Se, and Zn in 100% of the available herbal remedies (Obi et al., 2006). Toxic concentrations of heavy metals have been found in medicinal plants of Botswana and Kenya (Orisakwe et al., 2012; Paulsen et al., 2012). A major difference between presence of toxic metals in medicinal plants and which ends up in herbal formulations, and formulations in TCM and Ayurveda is that in the latter two (TCM and Ayurveda), heavy metals may be added intentionally for a perceived beneficial effect. In Ayurveda, heavy metals are added as ‘bhasma’ (calcined form), in which the metal is heated numerous times so that it ends up as a kind of soft, amorphous powder, which Ayurveda maintains is devoid of any toxic effects when mixed with herbal preparations (Sarkar, 2010).

Angong Niuhuang Pill contained Calculus Bovis (ox bezoar or dried gallstones of cattle), Buffalo horn extract, Moschus (musk deer), and Margarita (pearl) besides plants and minerals. Use of animal parts (the list can include birds, fish, reptiles, and insects) for therapeutic purposes (zootherapy) is a common practice in the traditional medicinal systems of many countries. The Xhosa and the Sotha communities of South Africa use a large number (only a few examples are given here) of reptiles (African rock python, puff adder) and animals (Chacma baboons, leopard, Cape porcupine) in their traditional medicinal formulations (Nieman et al., 2019). A survey in southern regions of Khyber Pakhtunkhwa, Pakistan documented 32 animal species for curing 37 types of diseases (Mussarat et al., 2021). The Saharia tribe of Rajasthan, India uses 15 animal species for 19 medicinal purposes (Mahawar and Jaroli, 2007). Calculus bovis reportedly contains eoxycorticosterone, methyl cholate, and biliverdin, which may be useful in the treatment of ischemic stroke (Liu et al., 2021). Water buffalo (*Bubali Cornu*) has been shown to have anti-pyretic effects in rats (Liu et al., 2016). Water buffalo and rhinoceros horns are attributed with anti-pyretic effects traditionally (But et al., 1990; But et al., 1991), but thus far any scientific evidences are lacking.

The use of Margarita (pearl) in traditional medicinal systems. In Ayurveda, pearl is referred to as a gem (ratna). Ayurveda mentions the use of Ruby (Maanikya), Pearl (Muktaa), Coral (Prawaala), Emerald (Taarkshya), Topaz (Pusparaaga), Diamond (Heeraka), Sapphire (Neela), Zircon (Gomeda), Cat’s Eye (Waidurya) in its various preparations along with herbal ingredients. Mukta Pisti is a strong Ayurvedic formulation in Ayurveda containing pearl, which is used for diarrhea with bleeding, heart disease, mania and psychosis, and bleeding disorders (Savrikar and Ravishankar, 2011). Khamira-e-Marwareed is a Unani herbal preparation containing *Mytilus margaritiferus* (pearl) and is used as a heart, brain, and nervine tonic, and palpitation and used during epidemics like COVID-19 (Khan et al., 2021). Although not the same as pearl, incinerated shells of large snails (*Turbinella pyrum*), known as ‘Shankha Bhasma’ is used in Ayurveda for treatment of hyperacidity, abdominal pain, and irritable bowel syndrome, as well as dysentery, gonorrhea, and jaundice (Chavan et al., 2018). Gemstones (including coral, pearl, and conches) have always been associated with zodiac signs and attributed to bringing luck (or the opposite) and/or ill health or good health depending on wearing the correct stone on the body in the form or ring, amulets or necklaces. However, whether they can play at all a healing role in diseases by themselves or in combination with herbs is an open question that needs to be solved.

A second TCM paper mentioned two formulations, Lian-Hua-Qing-Wen Capsule (LHQWC) and Jin-Hua-Qing-Gan Granule (JHQGG), both being recommended by the China Food and Drug Administration for the treatment of COVID-19 (Shi et al.). This is a review paper, which included data mining from other papers. One of the interesting anti-viral phytochemical mentioned in the paper is baicalin from *Scutellaria baicalensis* Georgi, which reportedly has shown virucidal activities against chikungunya virus (Oo et al., 2018) and Newcastle disease virus (Jia et al., 2016). Baicalin is also active against the respiratory syncytial virus and vesicular stomatitis virus. That baicalin has the potential of a novel broad-spectrum antiviral drug has been reviewed (Li et al., 2021). The flavonoid baicalin, derived from baicalein, has also been reported to be active against dengue virus 2 (DENV-2) by inhibiting viral replication (Moghaddam et al., 2014). Incidentally, the formulation JHQGG mentioned in this second TCM review also mentions *Lonicera japonica* as one of the ingredients.

The bioactive ingredients of the plant, *Lonicera japonica*, include chlorogenic acid, luteolin-7-*O*-β-D-glucopyranoside, and quercetin-3-*O*-β-D-glucopyranoside (Shang et al., 2011). Chlorogenic acid is considered a potential inhibitor of SARS-CoV-2; however, the conclusion is based on network pharmacology and molecular docking studies (Yu et al., 2020; Wang et al., 2022). Glycosyl flavonoids like luteolin-7-*O*-β-D-glucopyranoside and quercetin-3-*O*-β-D-glucopyranoside are considered therapeutic agents for COVID-19 (Godinho et al., 2021). The ingredients of the two formulations Lian-Hua-Qing-Wen Capsule (LHQWC) and Jin-Hua-Qing-Gan Granule (JHQGG) are mentioned by the authors to have broad spectrum anti-viral activity against 87 different types of viruses covering all the seven classes according to the Baltimore classification. This is a very important point. Conventional medicine has started to switch over from the paradigm of ‘one drug one disease’ to ‘repurposed drugs’ and ‘integrative medicine’, which by their very names are indicative of ‘one drug multiple diseases’. That a single formulation, plant or drug can be used for multiple diseases is an approach of traditional medicine, which needs to be endorsed by all.

The manifold effects of a single phytochemical, glycyrrhizic acid (GA), is illustrated very clearly in the paper authored by Sun et al. The compound GA is a major ingredient of licorice and is the most studied compound in licorice research for anti-viral activities (Ram, 2015; Gomaa and Abdel-Wadood, 2021). Besides anti-viral activities, GA is useful in COVID-19 patients with diabetes as a comorbidity (Orioli et al., 2020). Diabetes mellitus (DM) was found to be the third most prevalent comorbidity in COVID-19 patients after cardio-cerebrovascular disease and hypertension in China; DM can cause 2-3 fold increases in adverse outcomes in COVID-19 patients (Li Y. et al., 2020). Oxidative stress is increased in both DM and COVID-19 and both diseases share inflammatory pathways of disease progression (Paul et al., 2022); as such the two diseases can cause synergized aggravation in patients. GA have both antioxidant and anti-diabetic properties (Ming and Yin, 2013) and so can be beneficial in alleviation of DM and COVID-19.

The benefits of use of herbal formulations where the number of plants in the formulations go above one can be many. It enables the medicinal practitioner to adopt a holistic approach in the mode of treatment. Holistic means “treatment of the whole health”. Thus instead of confining the treatment to the disease, treatment takes into account the physical, mental, and social aspects of the patient, and different plants can be utilized to treat diverse aspects. Thus a given formulation can be changed both qualitatively and quantitatively with the addition or non-addition of a given plant and/or its quantity and dosage. The treatment is basically host-directed therapy (HDT), another name for it can be personalized medicine. For instance and again taking baicalin as an example, in addition to its virucidal properties, baicalin can balance host inflammatory response and limit immunopathologic injury (Pang et al., 2018), and baicalin, an aglycone of baicalin can inhibit production of interleukin-6 (IL-6) and interleukin-8 (IL-8) (Sithisarn et al., 2013). In response to COVID-19, a hyperinflammatory state can be produced in COVID-19 patients leading to high levels of pro-inflammatory cytokines, such as IL-1, IL-2, IL-6, TNF-α, IFN-γ, IP-10, GM-CSF, MCP-1, and IL-10, which can cause multiple organ damage and lead to severity of the disease (Zanza et al., 2022). So in this case, we can potentially have baicalin and baicalein for virucidal activity against SARS-CoV-2, alleviation of COVID-19 induced hyper-inflammatory response, and anti-diabetic treatment, diabetes being a major comorbidity in COVID-19 patients. Since GA has antioxidant properties, it can be beneficial for a host of other disorders including infectious diseases, cardiovascular disorders, and neurodegenerative disorders (Csányi and Miller, 2014; Ivanov et al., 2017; Singh et al., 2019), which increases oxidative stress and can be comorbidities in COVID-19 patients.

The final article in this series by Mahmud et al. dealt with integrative computational approach to identify inhibitors against SARS-CoV-2 main protease (Mpro). Mpro mediates viral replication and transcription, and is considered an attractive target for development of anti-SARS-CoV-2 drugs (Chunduru et al., 2021; Citarella et al., 2021; Sabbah et al., 2021; Shaji et al., 2021). Following a rigorous review of literature, the top three compounds were identified to be medicagol, faradiol, and flavanthrin. All three compounds bound to the active groove of SARS-CoV-2 Mpro and particularly the catalytic dyad consisting of amino acid residues His41 and Cys145. Medicagol (3-Hydroxy-8,9-methylenedioxycoumestan) is found in *Cicer chorassanicum* (Bge.) M. Popov. (Fabaceae) and *Sophora moorcroftiana* (Wall.) Benth. Ex Baker. (Fabaceae). Faradiol is a triterpenoid; faradiol myristate has been reported from *Calendula officinalis* L. (Asteraceae) and *Dittrichia viscosa* (L.) Greuter (Asteraceae). Flavanthrin, a dimeric 9,10-dihydrophenanthrene derivative has been reported from *Bulbophyllum reptans* (Lindl.) Lindl. (Orchidaceae) and *Eria lasiopetala* (Willd.) Ormerod (Orchidaceae). As such, all three compounds are present in natural sources and more specifically floral species.

A report published after publication of the paper being discussed mentioned that medicagol has high binding affinities for SARS-CoV-2 replicase polyprotein 1a, NSP16, and PL^pro^ (Roshni et al., 2022). Since this binding affinity extends to the recent Omicron variant of SARS-CoV-2, The Asteraceae and the Orchidaceae family plants may prove to be good reservoirs of anti-SARS-CoV-2 secondary metabolites. *In silico* and *in vitro* evidence suggests that faradiol can inhibit the NF-κB signaling pathway (Patil et al., 2015). SARS-CoV-2-induced cytokine storm and resultant severe inflammation has been linked to viral activation of NF-κB signaling pathway and production of pro-inflammatory cytokines (Hariharan et al., 2021). Flavanthrin has been reported from *Pholidota chinensis* Lindl. (Orchidaceae). Ethnic uses of the plant include chronic bronchitis. Pharmacological studies indicate antioxidant and anti-inflammatory properties (Nugraha et al., 2020).

So where does this issue and the articles published therein leave us? There is no question that the articles present a balanced view of the work done on COVID-19 and SARS-CoV-2. The reader becomes aware of potential therapeutics against COVID-19, the most promising among them coming from TCM. It is expected that researchers will be encouraged to amalgamate the various phytochemicals of anti-COVID-19 Chinese herbal formulations and come up with a therapeutic against this viral disease. It is also hoped for that other countries and continents will also mobilize resources to present a combined therapeutic effort against COVID-19 and other viral diseases.

## References

[B1] Agrawal P. Agrawal C. Blunden G. (2021). Rutin: A potential antiviral for repurposing as a SARS-CoV-2 main protease (Mpro) inhibitor. Nat. Prod. Commun. 16 (4), 1934578X2199172. 10.1177/1934578X21991723

[B2] Akhter J. Quéromès G. Pillai K. Kepenekian V. Badar S. Mekkawy A. H. (2021). The combination of bromelain and acetylcysteine (BromAc) synergistically inactivates SARS-CoV-2. Viruses 13, 425. 10.3390/v13030425 33800932 PMC7999995

[B3] Ali A. A. Milala M. A. Gulani I. A. (2015). Antimicrobial effects of crude bromelain extracted from pineapple fruit (*Ananas comosus (Linn.) Merr.*). Adv. Biochem. 3 (1), 1–4. 10.11648/j.ab.20150301.11

[B4] An X. Zhang Y. Duan L. Jin D. Zhao S. Zhou R. (2021). The direct evidence and mechanism of traditional Chinese medicine treatment of COVID-19. Biomed. Pharmacother. 137, 111267. 10.1016/j.biopha.2021.111267 33508618 PMC7836975

[B5] Andrews N. Stowe J. Kirsebom F. Toffa S. Rickeard T. Gallagher E. (2022). COVID-19 vaccine effectiveness against the Omicron (B.1.1.529) variant. N. Engl. J. Med. 386, 1532–1546. 10.1056/NEJMoa2119451 35249272 PMC8908811

[B6] Babili F. E. Lamade V. M. Fabre H. Charlot D. (2021). Reflection on medicinal plants, especially antivirals and how to reconsider ethnobotany as an interesting way for health preservation. Afr. J. Pharm. Pharmacol. 15 (1), 10–32. 10.5897/AJPP2020.5170

[B7] But P. P. Lung L. C. Tam Y. K. (1990). Ethnopharmacology of rhinoceros horn. I: Antipyretic effects of rhinoceros horn and other animal horns. J. Ethnopharmacol. 30 (2), 157–168. 10.1016/0378-8741(90)90005-e 2255207

[B8] But P. P. Tam Y. K. Lung L. C. (1991). Ethnopharmacology of rhinoceros horn. II: Antipyretic effects of prescriptions containing rhinoceros horn or water buffalo horn. J. Ethnopharmacol. 33 (1–2), 45–50. 10.1016/0378-8741(91)90159-B 1943172

[B9] Carlson C. J. Albery G. F. Merow C. Trisos C. H. Zipfel C. M. Eskew E. A. (2022). Climate change increases cross-species viral transmission risk. Nature 607 (7919), 555–562. 10.1038/s41586-022-04788-w 35483403

[B10] Chavan S. Tayade S. Gupta V. Deshmukh V. Sardeshmukh S. (2018). Pharmaceutical standardization and physicochemical characterization of traditional ayurvedic marine drug: Incinerated conch shell (*shankha bhasma*). Mar. Drugs 16 (11), 450. 10.3390/md16110450 PMC6266202 30445775

[B11] Chunduru K. Sankhe R. Begum F. Sodum N. Kumar N. Kishore A. (2021). *In silico* study to evaluate the antiviral activity of novel structures against 3C-like protease of novel coronavirus (COVID-19) and SARS-CoV. Med. Chem. 17 (4), 380–395. 10.2174/1573396316999200727125522 32720605

[B12] Citarella A. Scala A. Piperno A. Micale N. (2021). SARS-CoV-2 M^pro^: A potential target for peptidomimetics and small-molecule inhibitors. Biomolecules 11 (4), 607. 10.3390/biom11040607 33921886 PMC8073203

[B13] Conceição R. Nascimento C. Vascuncelos S. Saranraj P. Barreto A. Stephens P. (2020). History of medicinal plant use over time: From empirical knowledge to new guidelines in Brazil. J. Adv. Bio- Pharm. Pharmacovigil. 2 (1), 18–32. 10.5281/zenodo.3663389

[B14] Csányi G. Miller F. J. Jr (2014). Oxidative stress in cardiovascular disease. Int. J. Mol. Sci. 15, 6002–6008. 10.3390/ijms15046002 24722571 PMC4013610

[B15] de Koning A. P. J. Gu W. Castoe T. A. Batzer M. A. Pollock D. D. (2011). Repetitive elements may comprise over two-thirds of the human genome. PLoS Genet. 7 (12), e1002384. 10.1371/journal.pgen.1002384 22144907 PMC3228813

[B16] de S Leal E. Zanotto P. M. (2000). Viral diseases and human evolution. Mem. Inst. Oswaldo Cruz 95 (1), 193–200. 10.1590/s0074-02762000000700033 11142714

[B17] dos Santos Leite C. Bonafé G. A. Santos J. C. Martinez C. A. R. Ortega M. M. Ribeiro M. L. (2022). The anti-inflammatory properties of licorice (*Glycyrrhiza glabra*)-derived compounds in intestinal disorders. Int. J. Mol. Sci. 23, 4121. 10.3390/ijms23084121 35456938 PMC9025446

[B18] Dwarka D. Agoni C. Mellem J. J. Soliman M. E. Baijnath H. (2020). Identification of potential SARS-CoV-2 inhibitors from South African medicinal plant extracts using molecular modelling approaches. S. Afr. J. Bot. 133, 273–284. 10.1016/j.sajb.2020.07.035 32839635 PMC7437493

[B19] El-Sayed A. Kamel M. (2020). Climatic changes and their role in emergence and re-emergence of diseases. Environ. Sci. Pollut. Res. Int. 27 (18), 22336–22352. 10.1007/s11356-020-08896-w 32347486 PMC7187803

[B20] Godinho P. I. C. Soengas R. G. Silva V. L. M. (2021). Therapeutic potential of glycosyl flavonoids as anti-coronaviral agents. Pharmaceuticals 14, 546. 10.3390/ph14060546 34200456 PMC8227519

[B21] Gomaa A. A. Abdel-Wadood Y. A. (2021). The potential of glycyrrhizin and licorice extract in combating covid-19 and associated conditions. Phytomed. Plus. 1 (3), 100043. 10.1016/j.phyplu.2021.100043 35399823 PMC7886629

[B22] Gould E. (2009). Emerging viruses and the significance of climate change. Clin. Microbiol. Infect. 15 (6), 503. 10.1111/j.1469-0691.2009.02845.x 19604273

[B23] Hariharan A. Hakeem A. R. Radhakrishnan S. Reddy M. S. Rela M. (2021). The role and therapeutic potential of NF-kappa-B pathway in severe COVID-19 patients. Inflammopharmacology 29, 91–100. 10.1007/s10787-020-00773-9 33159646 PMC7648206

[B24] Hasan M. M. Rocha I. C. N. Ramos K. G. Cedeño T. D. D. dos Santos Costa A. C. Tsagkaris C. (2021). Emergence of highly infectious SARS-CoV-2 variants in Bangladesh: The need for systematic genetic surveillance as a public health strategy. Trop. Med. Health 49, 69. 10.1186/s41182-021-00360-w 34470674 PMC8408563

[B25] Islam M. R. (2022). The SARS-CoV-2 Omicron (B.1.1.529) variant and the re-emergence of COVID-19 in Europe: An alarm for Bangladesh. Health Sci. Rep. 5 (2), e545. 10.1002/hsr2.545 35308422 PMC8918917

[B26] Ivanov A. V. Bartosch B. Isaguliants M. G. (2017). Oxidative stress in infection and consequent disease. Oxid. Met. Cell. Longev. 2017, 3. 10.1155/2017/349604 PMC5309413 28255425

[B27] Ji S. Li Z. Song W. Wang Y. Liang W. Li K. (2016). Bioactive constituents of *Glycyrrhiza uralensis* (Licorice): Discovery of the effective components of a traditional herbal medicine. J. Nat. Prod. 79 (2), 281–292. 10.1021/acs.jnatprod.5b00877 26841168

[B28] Jia Y. Xu R. Hu Y. Zhu T. Ma T. Wu H. (2016). Anti-NDV activity of baicalin from a traditional Chinese medicine *in vitro* . J. Vet. Med. Sci. 78 (5), 819–824. 10.1292/jvms.15-0572 26902693 PMC4905837

[B29] Joshi M. D. (2014). *Glycyrrhiza glabra* (liquorice) – A potent medicinal herb. Int. J. Herb. Med. 2 (2), 132–136.

[B30] Kamala A. Middha S. K. Karigar C. S. (2018). Plants in traditional medicine with special reference to *Cyperus rotundus* L.: A review. 3 Biotech. 8, 309. 10.1007/s13205-018-1328-6 PMC6037646 30002998

[B31] Khan F. Ansari A. N. Nayab M. (2021). Rationalistic approach in COVID-19 prevention through intervention of Unani medicine prevalent in epidemic – a review. J. Herb. Med. 30, 100515. 10.1016/j.hermed.2021.100515 34722133 PMC8547781

[B32] Li B. Yang J. Zhao F. Zhi L. Wang X. Liu L. (2020). Prevalence and impact of cardiovascular metabolic diseases on COVID-19 in China. Clin. Res. Cardiol. 109 (5), 531–538. 10.1007/s00392-020-01626-9 32161990 PMC7087935

[B33] Li K. Liang Y. Cheng A. Wang Q. Li Y. Wei H. (2021). Antiviral properties of baicalin: A concise review. Rev. Bras. Farmacogn. 31 (4), 408–419. 10.1007/s43450-021-00182-1 34642508 PMC8493948

[B34] Li Y. Li W. Fu C. Song Y. Fu Q. (2020). *Lonicerae japonicae* flos and lonicerae flos: A systematic review of ethnopharmacology, phytochemistry and pharmacology. Phytochem. Rev. 19, 1–61. 10.1007/s11101-019-09655-7 32206048 PMC7088551

[B35] Liu F. Li L. Chen J. Wu Y. Cao Y. Zhong P. (2021). A network pharmacology to explore the mechanism of *Calculus Bovis* in the treatment of ischemic stroke. Biomed. Res. Int. 2021, 6611018. 10.1155/2021/6611018 33778069 PMC7972848

[B36] Liu R. Huang Q. Shan J. Duan J.-A. Zhu Z. Liu P. (2016). Metabolomics of the antipyretic effects of bubali Cornu (water buffalo horn) in rats. PLoS One 11 (7), e0158478. 10.1371/journal.pone.0158478 27384078 PMC4934856

[B37] Luo L. Wang Bo. Jiang J. Fitzgerald M. Huang Q. Yu Z. (2020). Heavy metal contaminations in herbal medicines: Determination, comprehensive risk assessments, and solutions. Front. Pharmacol. 11, 595335. 10.3389/fphar.2020.595335 33597875 PMC7883644

[B38] Magouras I. Brookes V. J. Jori F. Martin A. Pfeiffer D. U. Dürr S. (2020). Emerging zoonotic diseases: Should we rethink the animal-human interface? Front. Vet. Sci. 7, 582743. 10.3389/fvets.2020.582743 33195602 PMC7642492

[B39] Mahawar M. M. Jaroli D. P. (2007). Traditional knowledge on zootherapeutic uses by the Saharia tribe of Rajasthan, India. J. Ethnobiol. Ethnomed. 3, 25. 10.1186/1746-4269-3-25 17547781 PMC1892771

[B41] Ming L. J. Yin A. C. Y. (2013). Therapeutic effects of glycyrrhizic acid. Nat. Prod. Commun. 8 (3), 415–418. 10.1177/1934578x1300800335 23678825

[B42] Moghaddam E. Teoh B.-T. Sam S.-S. Lani R. Hassandarvish P. Chik Z. (2014). Baicalin, a metabolite of baicalein with antiviral activity against dengue virus. Sci. Rep. 4, 5452. 10.1038/srep05452 24965553 PMC4071309

[B43] Moumen A. Azizi G. Chekroun K. B. Baghour M. (2016). The effects of livestock methane emission on the global warming: A review. Int. J. Glob. Warm. 9 (2), 229–253. 10.1504/IJGW.2016.074956

[B44] Mussarat S. Ali R. Ali S. Mothana R. A. Ullah R. Adnan M. (2021). Medicinal animals and plants as alternative and complementary medicine in southern regions of khyber Pakhtunkhwa, Pakistan. Front. Pharmacol. 12, 649046. 10.3389/fphar.2021.649046 34504421 PMC8422074

[B45] Najmi A. Javed S. A. Bratty M. A. Alhazmi H. A. (2022). Modern approaches in the discovery and development of plant-based natural products and their analogues as potential therapeutic agents. Molecules 27 (2), 349. 10.3390/molecules27020349 35056662 PMC8779633

[B46] Nieman W. A. Leslie A. J. Wilkinson A. (2019). Traditional medicinal animal use by Xhosa and Sotho communities in the Western Cape Province, South Africa. J. Ethnobiol. Ethnomed. 19, 34. 10.1186/s13002-019-0311-6 PMC6617652 31288841

[B47] Nugraha A. S. Triatmoko B. Wangchuk P. Keller P. A. (2020). Vascular epiphytic medicinal plants as sources of therapeutic agents: Their ethnopharmacological uses, chemical composition, and biological activities. Biomolecules 10 (2), 181. 10.3390/biom10020181 PMC7072150 31991657

[B48] Obi E. Akunyili D. N. Ekpo B. Orisakwe O. E. (2006). Heavy metal hazards of Nigerian herbal remedies. Sci. Total Environ. 369 (1-3), 35–41. 10.1016/j.scitotenv.2006.04.024 16759683

[B50] Onken J. E. Greer P. K. Calingaert B. Hale L. P. (2008). Bromelain treatment decreases secretion of pro-inflammatory cytokines and chemokines by colon biopsies *in vitro* . Clin. Immunol. 126 (3), 345–352. 10.1016/j.clim.2007.11.002 18160345 PMC2269703

[B51] Oo A. Rausalu K. Merits A. Higgs S. Vanlandingham D. Bakar S. A. (2018). Deciphering the potential of baicalin as an antiviral agent for chikungunya virus infection. Antivir. Res. 150, 101–111. 10.1016/j.antiviral.2017.12.012 29269135

[B52] Orioli L. Hermans M. P. Thissen J. P. Maiter D. Vandeleene B. Yombi J. C. (2020). COVID-19 in diabetic patients: Related risks and specifics of management. Ann. Endocrinol. Paris. 81 (2-3), 101–109. 10.1016/j.ando.2020.05.001 32413342 PMC7217100

[B87] Orisakwe O. E. Kanayochukwu N. J. Nwadiuto A. C. Daniel D. Onyinyechi O. (2012). Evaluation of potential dietary toxicity of heavy metals of vegetables. J. Environ. Analyt. Toxicol. 2, 3. 10.4172/2161-0525.1000136

[B53] Pang P. Zheng K. Wu S. Xu H. Deng L. Shi Y. (2018). Baicalin downregulates RLRs signaling pathway to control influenza A virus infection and improve the prognosis. Evid. Based. Complement. Altern. Med. 2018, 4923062. 10.1155/2018/4923062 PMC5846362 29681974

[B54] Patil K. R. Mohapatra P. Patel H. M. Goyal S. N. Ojha S. Kundu C. N. (2015). Pentacyclic terpenoids inhibit IKKβ mediated activation of NF-κB pathway: *In silico* and *in vitro* evidences. PLoS One 10 (5), e0125709. 10.1371/journal.pone.0125709 25938234 PMC4418667

[B55] Patz Z. A. Epstein P. R. Burke T. A. Balbus J. M. (1996). Global climate change and emerging infectious diseases. JAMA J. Am. Med. Assoc. 275 (3), 217–223. 10.1001/jama.275.3.217 8604175

[B56] Paul A. K. Hossain M. K. Mahboob T. Nissapatorn V. Wilairatana P. Jahan R. (2022). Does oxidative stress management help alleviation of COVID-19 symptoms in patients experiencing diabetes? Nutrients 14 (2), 321. 10.3390/nu14020321 35057501 PMC8780958

[B88] Paulsen M. Gatebe E. Gitu L. (2012). Profile of heavy metals in selected medicinal plants used for the treatment of diabetes, malaria and pneumonia in Kisii region, Southwest Kenya Global J. Pharmacol. 6, 245–251. 10.1080/15569543.2016.1225768

[B57] Pohjala L. Tammela P. (2012). Aggregating behavior of phenolic compounds—A source of false bioassay results? Molecules 17, 10774–10790. 10.3390/molecules170910774 22960870 PMC6268869

[B58] Popoola T. D. Segun P. A. Ekuadzi E. Dickson R. A. Awotona O. R. Nahar L. (2022). West African medicinal plants and their constituent compounds as treatments for viral infections, including SARS-CoV-2/COVID-19. Daru. 30 (1), 191–210. 10.1007/s40199-022-00437-9 35476297 PMC9043090

[B59] Ram S. (2015). A bibliometric assessment of liquorice (*Glycyrrhiza glabra*) research trends. Ann. Libr. Inf. Stud. 62 (1), 27–32. http://hdl.handle.net/123456789/31613.

[B60] Rasamiravaka T. Kahumba J. Okusa P. N. Amuri B. Bizumukama L. Kalonji J.-B. N. (2015). Traditional African medicine: From ancestral knowledge to a modern integrated future. Science 350 (6262), S61–S63.

[B61] Roshni J. Vaishali R. Ganesh K. S. Dharani N. Alzahrani K. J. Banjer H. J. (2022). Multi-target potential of Indian phytochemicals against SARS-CoV-2: A docking, molecular dynamics and MM-GBSA approach extended to Omicron B.1.1.529. J. Infect. Public Health 15 (6), 662–669. 10.1016/j.jiph.2022.05.002 35617830 PMC9101941

[B62] Russo M. Moccia S. Spagnuolo C. Tedesco I. Russo G. L. (2020). Roles of flavonoids against coronavirus infection. Chem. Biol. Interact. 328, 109211. 10.1016/j.cbi.2020.109211 32735799 PMC7385538

[B63] Sabbah D. A. Hajjo R. Bardaweel S. K. Zhong H. A. (2021). An updated review on SARS-CoV-2 main proteinase (MPro): Protein structure and small-molecule inhibitors. Curr. Top. Med. Chem. 21 (6), 442–460. 10.2174/1568026620666201207095117 33292134

[B64] Sarkar P. K. (2010). Ayurvedic bhasma: The most ancient application of nanomedicine. J. Sci. Ind. Res. 69 (12), 901–905.

[B65] Savrikar S. S. Ravishankar B. (2011). Introduction to ‘rasashaastra’ the iatrochemistry of Ayurveda. Afr. J. Tradit. Complement. Altern. Med. 8 (5), 66–82. 10.4314/ajtcam.v8i5S.1 PMC3252715 22754059

[B66] Schvoerer E. Massue J.-P. Gut J.-P. Stoll-Keller F. (2008). Climate change: Impact on viral diseases. Open Epidemiol. J. 1 (1), 53–56. 10.2174/1874297100801010053

[B67] Shaji D. Yamamoto S. Saito R. Suzuki R. Nakamura S. Kurita N. (2021). Proposal of novel natural inhibitors of severe acute respiratory syndrome coronavirus 2 main protease: Molecular docking and *ab initio* fragment molecular orbital calculations. Biophys. Chem. 275, 106608. 10.1016/j.bpc.2021.106608 33962341 PMC8084281

[B68] Shang X. Pan H. Li M. Miao X. Ding H. (2011). *Lonicera japonica* Thunb.: Ethnopharmacology, phytochemistry and pharmacology of an important traditional Chinese medicine. J. Ethnopharmacol. 138 (1), 1–21. 10.1016/j.jep.2011.08.016 21864666 PMC7127058

[B69] Sharma R. Martins N. Kuca K. Chaudhary A. Kabra A. Rao M. M. (2019). Chyawanprash: A traditional Indian bioactive health supplement. Biomolecules 9 (5), 161. 10.3390/biom9050161 PMC6571565 31035513

[B70] Sharp P. M. Simmond S. P. (2011). Evaluating the evidence for virus/host co-evolution. Curr. Opin. Virol. 1 (5), 436–441. 10.1016/j.coviro.2011.10.018 22440848

[B71] Shawan M. M. A. K. Halder S. K. Hasan M. A. (2021). Luteolin and abyssinone II as potential inhibitors of SARS-CoV-2: An *in silico* molecular modeling approach in battling the COVID-19 outbreak. Bull. Natl. Res. Cent. 45 (1), 27. 10.1186/s42269-020-00479-6 33495684 PMC7816153

[B72] Singh A. Kukreti R. Saso L. Kukreti S. (2019). Oxidative stress: A key modulator in neurodegenerative diseases. Molecules 24, 1583. 10.3390/molecules24081583 PMC6514564 31013638

[B73] Sithisarn P. Michaelis M. Schubert-Zsilavecz M. Cinatl J. Jr (2013). Differential antiviral and anti-inflammatory mechanisms of the flavonoids biochanin A and baicalein in H_5_N_1_ influenza A virus-infected cells. Antivir. Res. 97 (1), 41–48. 10.1016/j.antiviral.2012.10.004 23098745

[B74] Skidmore M. E. Moffette F. Rausch L. Christie M. Munger J. Gibbs H. K. (2021). Cattle ranchers and deforestation in the Brazilian Amazon: Production, location, and policies. Glob. Environ. Change 68, 102280. 10.1016/j.gloenvcha.2021.102280

[B75] Solnier J. Fladerer J.-P. (2021). Flavonoids: A complementary approach to conventional therapy of COVID-19? Phytochem. Rev. 20 (4), 773–795. 10.1007/s11101-020-09720-6 32982616 PMC7500502

[B76] Tallei T. E. Adam A. A. Elseehy M. M. El-Shehawi A. M. Mahmoud E. A. Tania A. D. Fatimawali (2022). Fruit bromelain-derived peptide potentially restrains the attachment of SARS-CoV-2 variants to hACE2: A pharmacoinformatics approach. Molecules 27, 260. 10.3390/molecules27010260 35011492 PMC8746556

[B77] Tariq M. (2015). An overview of deforestation causes and its environmental hazards in Khyber Pukhtunkhwa. J. Nat. Sci. Res. 5 (1), 52–57.

[B78] Umar H. I. Josiah S. S. Saliu T. P. Jimoh T. O. Ajayi A. Danjuma J. B. (2021). *In-silico* analysis of the inhibition of the SARS-CoV-2 main protease by some active compounds from selected African plants. J. Taibah Univ. Med. Sci. 16 (2), 162–176. 10.1016/j.jtumed.2020.12.005 33437230 PMC7787523

[B79] Vora N. (2008). Impact of anthropogenic environmental alterations on vector-borne diseases. Medscape J. Med. 10 (10), 238. 19099032 PMC2605134

[B80] Wang W.-X. Zhang Y.-R. Luo S.-Y. Zhang Y.-S. Zhang Y. Tang C. (2022). Chlorogenic acid, a natural product as potential inhibitor of COVID-19: Virtual screening experiment based on network pharmacology and molecular docking. Nat. Prod. Res. 36 (10), 2580–2584. 10.1080/14786419.2021.1904923 33769143

[B81] WHO (2022a). Disease outbreak news; multi-country monkeypox outbreak in non-endemic countries: Update. Available at: https://www.who.int/emergencies/disease-outbreak-news/item/2022-DON390 (Accessed June 19, 2022).

[B82] WHO (2022b). WHO recommends highly successful COVID-19 therapy and calls for wide geographical distribution and transparency from originator. Available at: https://www.who.int/news/item/22-04-2022-who-recommends-highly-successful-covid-19-therapy-and-calls-for-wide-geographical-distribution-and-transparency-from-originator (Accessed June 19, 2022).

[B83] Wolfe N. D. Daszak P. Kilpatrick A. M. Burke D. S. (2005). Bushmeat hunting, deforestation, and prediction of zoonoses emergence. Emerg. Infect. Dis. 11, 1822–1827. 10.3201/eid1112.040789 16485465 PMC3367616

[B84] Yu J. W. Wang L. Bao L. D. (2020). Exploring the active compounds of traditional Mongolian medicine in intervention of novel coronavirus (COVID-19) based on molecular docking method. J. Funct. Foods 71, 104016. 10.1016/j.jff.2020.104016 32421102 PMC7225714

[B85] Zanza C. Romenskaya T. Manetti A. C. Franceschi F. Russa R. L. Bertozzi G. (2022). Cytokine storm in COVID-19: Immunopathogenesis and therapy. Medicina 58, 144. 10.3390/medicina58020144 35208467 PMC8876409

[B86] Zhao J. Yang J. Xie Y. (2019). Improvement strategies for the oral bioavailability of poorly water-soluble flavonoids: An overview. Int. J. Pharm. 570, 118642. 10.1016/j.ijpharm.2019.118642 31446024

